# Segmentation of White Blood Cell from Acute Lymphoblastic Leukemia Images Using Dual-Threshold Method

**DOI:** 10.1155/2016/9514707

**Published:** 2016-05-22

**Authors:** Yan Li, Rui Zhu, Lei Mi, Yihui Cao, Di Yao

**Affiliations:** ^1^State Key Laboratory of Transient Optics and Photonics, Xi'an Institute of Optics and Precision Mechanics of Chinese Academy of Sciences, Xi'an 710119, China; ^2^University of Chinese Academy of Sciences, 52 Sanlihe Road, Beijing 100864, China; ^3^Shenzhen Vivolight Medical Device and Technology Co., Ltd., Shenzhen 518000, China

## Abstract

We propose a dual-threshold method based on a strategic combination of RGB and HSV color space for white blood cell (WBC) segmentation. The proposed method consists of three main parts: preprocessing, threshold segmentation, and postprocessing. In the preprocessing part, we get two images for further processing: one contrast-stretched gray image and one H component image from transformed HSV color space. In the threshold segmentation part, a dual-threshold method is proposed for improving the conventional single-threshold approaches and a golden section search method is used for determining the optimal thresholds. For the postprocessing part, mathematical morphology and median filtering are utilized to denoise and remove incomplete WBCs. The proposed method was tested in segmenting the lymphoblasts on a public Acute Lymphoblastic Leukemia (ALL) image dataset. The results show that the performance of the proposed method is better than single-threshold approach independently performed in RGB and HSV color space and the overall single WBC segmentation accuracy reaches 97.85%, showing a good prospect in subsequent lymphoblast classification and ALL diagnosis.

## 1. Introduction

Automatic white blood cell segmentation which plays an important role in automatic blood cell morphology analysis remains a challenging issue because of the morphological diversity of WBCs and the complex background of blood microscopic images. In this paper, our focus is the segmentation of lymphoblast—one kind abnormal white blood cell from Acute Lymphoblastic Leukemia images for ALL classification.

Acute Lymphocytic Leukemia, also known as Acute Lymphoblastic Leukemia, is a serious hematic disease characterized by the overproduction and continuous multiplication of malignant and immature WBCs (referred to as lymphoblasts or blasts). It is fatal if left untreated due to blasts' rapid spread into the bloodstream and other vital organs. Fortunately, early diagnosis of the disease is helpful and beneficial to the recovery of patients, especially in the case of children [1].

**Figure 1:**
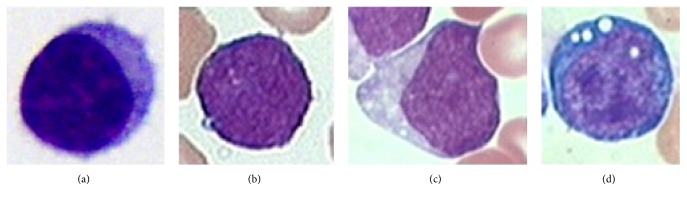
Morphological variability associated with the blast cells according to the FAB classification. (a) Healthy lymphocytes cell from non-ALL patients. (b–d) Lymphoblasts from ALL patients where (b), (c), and (d) are L1, L2, and L3, respectively.

According to the FAB (French-American-British) classification standard built on blast cell's shape and pattern variability, ALL can be classified into three types: L1, L2, and L3 (Figures 1(b)–1(d)) [2]. Accurate classification can offer doctors useful information for therapeutic plan selection and follow-up work. The microscope inspection of blood smears by counting different kinds of WBCs is one of the most frequently performed blood tests by hematologists [3]. For decades, the operation is performed by experienced operators. However, this is a time-consuming and tedious work. In addition, diagnosis results always tend to be subjective and imprecise and also are difficult to be reproduced afterwards.

As a substitute, automatic identification technology of WBCs known for low-cost, homogenous accuracy has gained more and more focus in domain of hematic related disease diagnosis. In general, the system consists of four parts: WBC segmentation, feature extraction, classification, and counting. Since segmentation results directly influence the accuracy of classification and counting, it has become a very hot topic in clinical diagnosis [4].

Some work on microscopic WBC image segmentation is available in the literature [3–25]. Methods can be classified into three types: threshold-based methods, pattern recognition-based methods, and deformable model-based methods. Threshold-based methods include Otsu's method [4–6], the region growing method [7], watershed method [8–10], and their combination [11]. Cseke [4] presented a fast segmentation scheme with automatic thresholding where thresholds are selected with a simple recursive method derived from maximizing the interclass variance between dark, gray, and bright regions based on the method proposed by Otsu. The method works well for nucleus and background segmentation. However, it cannot separate cytoplasm from the red blood cells. Wu et al. [6] developed an iterative Otsu's threshold approach based on circular histogram for the leukocyte segmentation using H&S components of HSI model. Experimental results show that the method works successfully in the segmentation of WBC nucleus but loses the cytoplasm information. Dorini et al. [11] divided the WBC segmentation process into two steps. In the first step, they extracted the cell nucleus using the watershed transform by Image Forest Transform (IFT). Then, they segmented the WBC cytoplasm using basic operations such as thresholding and morphological opening via the size distribution information of the RBC, but the method tends to produce oversegmentation in presence of noise.

As leukocytes in microscopic images can be treated as objects, pattern recognition methods are also used to perform the segmentation which can be categorized as supervised or unsupervised [12]. Supervised methods classify the objects using learning-based approaches such as Support Vector Machine (SVM) [13] and Artificial Neural Network (ANN) while unsupervised methods also known as clustering methods mainly including *k*-means clustering [14–16], fuzzy *C*-means [17], and expectation-maximization extract the objects from the data itself. In [13], Guo et al. proposed multispectral imaging techniques with a spectral calibration method to acquire device-independent images and then applied SVM directly to the spectrum of each pixel to segment the whole microscopic image into four types of regions: nucleus, cytoplasm, erythrocytes, and background. Segmentation results are satisfactory but the implementation speed needs to be boosted.

Deformable model-based methods can be classified into parametric models and geometric models based on contour representation. Besides level set method [9], the active contour model also known as a snake is the most common [18, 19]. In [18], Ko et al. introduced a new WBC image segmentation method using stepwise merging rules based on mean-shift clustering and boundary removal rules with a gradient vector flow (GVF) snake. Removal rules are used to remove the boundary and noise edges while a GVF snake is forced to deform to the cytoplasm boundary edges. Due to a weak difference between the cytoplasm and the background or contact with RBCs, some experimental results were slightly oversegmented. Other segmentation methods for WBCs aside from the above three categories are morphological operations [3, 11, 20], hybrid methods [9, 10, 19, 21], and so on. Hybrid methods combine two or more methods mentioned above to achieve better results, such as combination of a Lab color space based segmentation method and a gray level thresholding method [21] and combination of an Otsu method and an active contour method [19].

**Figure 2:**
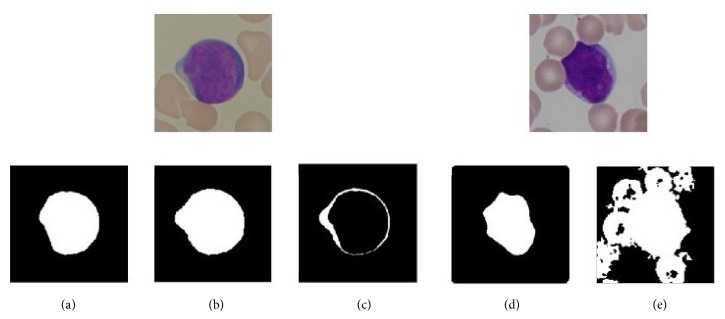
Background of dual-threshold method. (a, d) Segmentation results of single-threshold method based on RGB color space or S channel image from HSV color space (only nucleus is extracted). (b) Segmentation result of uniform image using single-threshold method based on H channel image of HSV color space (nucleus and cytoplasm are all extracted). (c) Expected segmentation result of cytoplasm. (e) Segmentation result of image having illumination variations by single-threshold method based on H channel image of HSV color space (much noise exists in segmentation result).

Generally, the ultimate goal of WBC segmentation is to extract whole WBC from a complicated background and segment every WBC into morphological components such as nucleus and cytoplasm. Among the methods mentioned above, RGB color space based threshold approaches are most widely used due to their high efficiency and reliability. However, cytoplasm has a big variance of color and its color is quite similar to image background. Thus, in many previous works, gray image based threshold methods are only utilized to segment nucleus (Figures 2(a) and 2(d)). As to cytoplasm extraction (Figure 2(c)), other auxiliary segmentation schemes are needed [19]. What is more, aside from single-threshold-based segmentation methods, nucleus and cytoplasm are, respectively, segmented with different methods in many other segmentation schemes too [3, 8, 9, 11, 18]. To facilitate cytoplasm segmentation via threshold-based methods, some researchers have turned their attention to segmenting in transformed color space such as HSV [22, 23], HSI [24], and Lab [21]. As an example, Eldahshan et al. [22] proposed to segment WBC from its background using hue channel of HSV color space based single-threshold method. The results show that the proposed framework works well for uniform images (Figure 2(b)) but inconsistent for the images having illumination variations (Figure 2(e)).

In this paper, we propose a general method to segment WBC (both nucleus and cytoplasm are included) from image background regardless of illumination variations by effectively combining the RGB color space based single-threshold method and HSV color space based single-threshold method to construct a new technique, which is named dual-threshold method to overcome the weakness of the component methods. In this way, cytoplasm can further be segmented by subtracting nucleus which can be obtained through various intuitive methods such as Otsu's method [6] and *k*-means clustering [14] from the above result easily. Though, it is not included in this paper. To determine the value of the two thresholds, golden section search method is selected. At last, except qualitative assessment, we also make a quantitative comparison of our method with two single-threshold-based methods, respectively, implemented in RGB and HSV color space [23] in terms of a widely used evaluation metric DSC in image segmentation field.

The rest of this paper is organized as follows. In Section 2, we elaborate the complete methodology of the dual-threshold algorithm. In Section 3, we describe an evaluation of this method in terms of its accuracy and robustness. In Section 4, concluding remark is given.

## 2. Materials and Methods

### 2.1. Overview of the Approach

**Figure 3:**
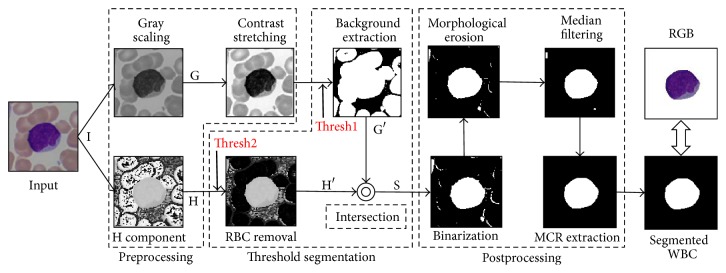
Flowchart of the proposed dual-threshold segmentation scheme.

The proposed dual-threshold method consists of three phases: preprocessing, threshold segmentation, and postprocessing.  Figure 3 presents an overview of the proposed approach. In the preprocessing phase, we get two images for further processing: one contrast-stretched gray image and one H component image from transformed HSV color space. The threshold segmentation phase consists of three main steps: image background extraction, red blood cell separation, and the optimal threshold selection. In the background extraction step, RGB color space based single-threshold method is employed while, in red blood cell separation step, H channel image based single-threshold method is utilized. For the optimal threshold selection part, golden section search method is used. Finally, in the postprocessing phase, mathematical morphology and median filtering are utilized to denoise and remove incomplete WBCs. The whole process can be summarized as the following five steps. The details of each step are given in the following subsections.

**Step 1 .**

Given an input image I, get its gray-scaling image G as well as H component image H from transformed HSV color space.

**Step 2 .**

Do contrast stretching operation to G, and then use threshold Thresh1 to extract image background which is shown in black in G′ (Figure 3).

**Step 3 .**

Through threshold Thresh2, separate the red blood cells from H (resultant image is H′).

**Step 4 .**

Get a gray image S by intersecting G′ with H′.

**Step 5 .**

Binarization, morphological erosion, and median filtering are performed on S followed by maximum connected region (MCR) extraction to get a binary image, fill small holes, connect narrow gaps, and remove small dots as well as incomplete WBCs in the image.

### 2.2. Preprocessing

**Figure 4:**
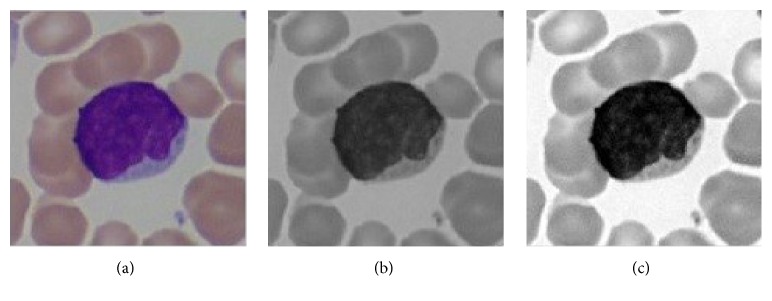
Global contrast stretching. (a) Original image. (b) Gray image. (c) Contrast-stretched image.

In this step, two roughly processed images are obtained for further processing. At first, we convert the color image into a gray one (Figure 4(b)). At the same time, transform the source RGB color space into HSV color space, and then extract H channel image denoted by H in Figure 3 from HSV color space. From Figure 4, we can see that the contrast between foreground and background pixels in the grayscale is typically not sufficient to classify the pixels precisely. To increase the contrast of the image, the global contrast stretching (GCS) technique which can spread out the range of scene illumination is employed. GCS is performed by sliding a window (called the KERNEL) across the image and adjusting the center element using the following formula [25]:

(1) $$\begin{array}{c} I_{p}\left(\;x,y\;\right)=\frac{255•\left[I_{o}\left(x,y\right)-min\right]}{\left(max-min\right)}, \end{array}$$

where  (*x*, *y*) is the coordinate of the image pixel; *I*_*p*_(*x*, *y*) is the output color level for the pixel (*x*, *y*) from the contrast stretching process; *I*_*o*_(*x*, *y*) is the input color level for the pixel (*x*, *y*); min and max are the minimum and maximum color level value in the input image.

After GCS (Figure 4(c)), we can see that the contrast throughout the image is equalized and it has been easier to see the image details in the regions that are originally very obscure. More importantly, contrast between foreground and background pixels is greatly enhanced which is supposed to facilitate image background extraction via single-threshold method which will be clarified in the next part.

### 2.3. Threshold Segmentation

#### 2.3.1. Image Background Extraction

**Figure 5:**
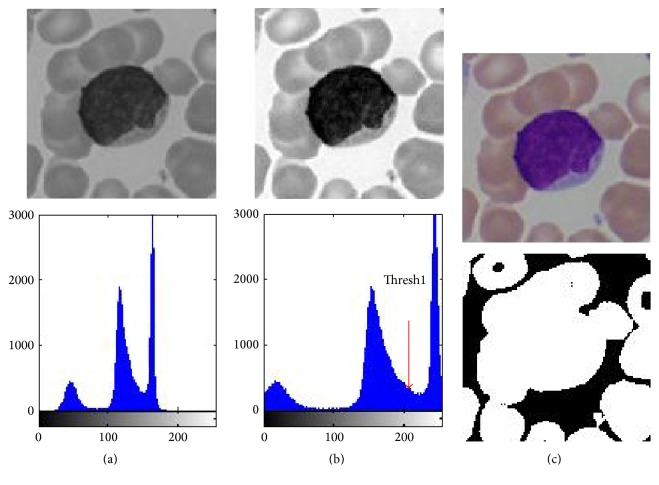
Image background extraction through gray image based single-threshold method. (a) Original gray image and its histogram. (b) Contrast-stretched image and its histogram. (c) Original color image and resultant binary image after background extraction (the extracted background is shown in black in (c)).

From Figure 2(e), it can be seen that noise in final segmentation result mainly comes from two aspects: background and red blood cells. The following two steps background extraction and red blood cell separation are all somewhat based on this fact. In Figure 5, histogram of the contrast-stretched gray image presents a triple-modal, respectively, representing white blood cell nucleus, red blood cell (cytoplasm), and background. After GCS, background now has a certain contrast with other components in the image, so it is feasible to extract it through single-threshold (marked as Thresh1 in Figure 5) method. However, as it has been stated in Introduction, it is still difficult to separate the whole white blood cell (both nucleus and cytoplasm included) from the image in that cytoplasm has a similar gray intensity with red blood cells. So the first stage of our method is to extract the image background shown in black in Figure 5(c).

#### 2.3.2. Red Blood Cell Separation

In Section 2.3.1, image background has been extracted. If we can continue finding an effective method to remove red blood cells in the image, WBC can be segmented easily by subtracting background and red blood cells from the original image. As a result, our goal in this step is to separate the red blood cells from the image.

The purpose of a color space is to facilitate the specification of colors in some standard [26]. A color space is typically represented by a three- or four-dimension matrix in mathematics, such as RGB, HSV, Lab, and CMYK. The RGB color space is the most common color space used in electronic devices. In this color space, each color can be obtained by the addition of three primary colors: red, green, and blue. Generally, the original stained blood smear image is represented by the RGB color space in the RGB model. The HSV color space has three components: hue (H), saturation (S), and value (V). Hue represents color. In this model, it is an angle from 0 to 360 degrees. Saturation indicates the range of gray in the color space. It ranges from 0 to 100%. Sometimes the value is calculated from 0 to 1. When the value is “0,” the color is gray and when the value is “1,” the color is a primary color. A faded color is due to a lower saturation level, which means the color contains more gray. Value is the brightness of the color and varies with color saturation. It ranges from 0 to 100%. When the value is “0,” the color space will be totally black. With the increase in the value, the color space brightness is up and shows various colors. The HSV color space is quite similar to the way in which humans perceive color. The colors used in this space can be clearly defined by human perception, which is not always the case with RGB. These characteristics make the HSV color space more suitable for image segmentation and analysis than the RGB model. In Lab color space, L defines lightness, a denotes red/green value, and b represents the yellow/blue value [21]. It is known as device independent, meaning the Lab color space can communicate different colors across different devices. The above color spaces can be converted into each other according to related formulas.

**Figure 6:**
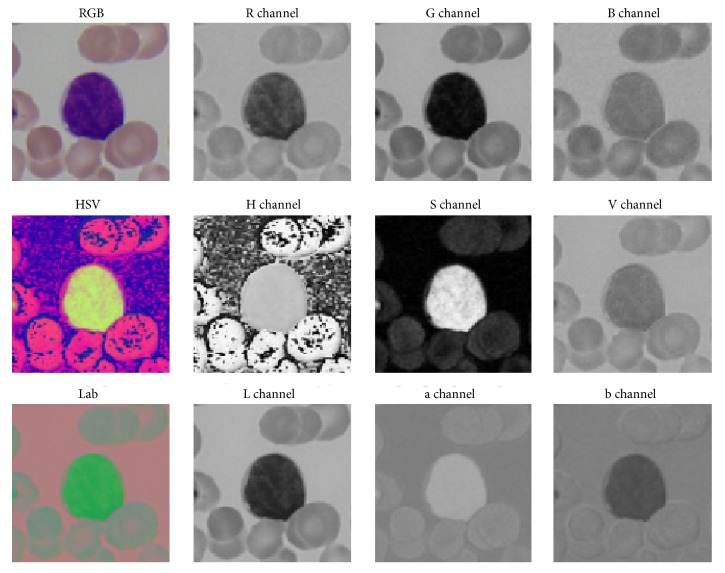
Images in RGB, HSV, and Lab color spaces and their component images (R, G, B; H, S, V; and L, a, b).

**Figure 7:**
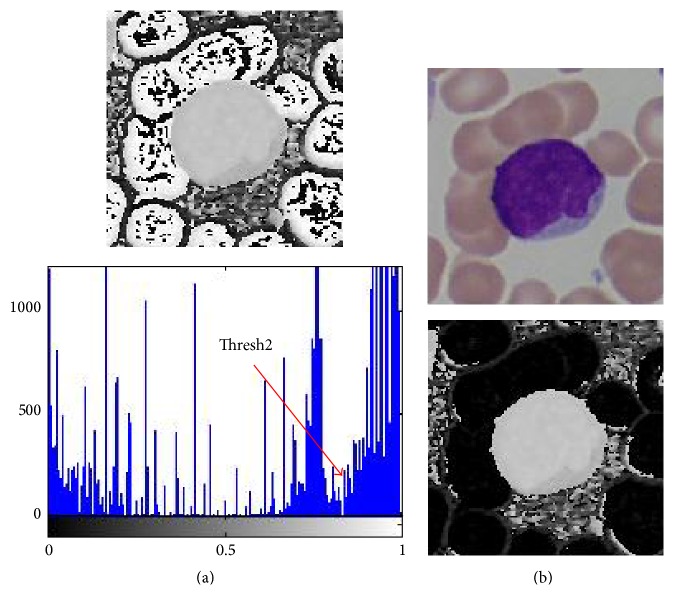
Red blood cell removal through H channel image based single-threshold method. (a) H channel image and its histogram. (b) Original image and the H channel image after red blood cell removal.

In Figure 6, a comparison of blood cell image, respectively, in RGB, HSV, and Lab color spaces, and each channel image is given. From the figure, it can be observed that, in most single-channel images (such as G-, S-, L-, and b-channel), cytoplasm has a similar gray intensity with the background and red blood cells, making it difficult to be separated from red blood cells and background through Otsu's method except H channel image where cytoplasm has a similar gray intensity with nucleus and a certain contrast with red blood cells. Therefore, to avoid cytoplasm being removed together with red blood cells (which is the case in G-channel, S-channel, and some other channels), H channel image is selected for red blood cell removal in this step. In Figure 7(a), H channel image and its histogram are given. We can see that, in the image, red blood cells are the brightest part. So we can remove them through a suitable threshold marked as Thresh2 in the figure.  Figure 7(b) shows the resultant image after red blood cell separation.

#### 2.3.3. Threshold Selection

The proposed algorithm has two parameters, Thresh1 and Thresh2, which are, respectively, used in image background extraction and red blood cell removal steps of the white blood cell segmentation. From the histograms of contrast-stretched gray image (Figure 5(b)) and H channel image (Figure 7(a)), we can see that the ranges of Thresh1 and Thresh2 can be roughly estimated through a priori knowledge. But in concrete algorithm implementation, randomly selected thresholds even when they are in the reasonable scope just cannot guarantee any kind of optimality. So we formulate threshold selection as an optimization problem with two variables (Thresh1 and Thresh2) and tend to use an appropriate method to find the optimal solution.

To formulate a proper objective function *f*, let us introduce Dice Similarity Coefficient (DSC) first, which is usually used to evaluate segmentation effect quantitatively. In the next section, we will use it for quantitative comparison of the proposed method and two other segmentation schemes. It is defined as

(2) $$\begin{array}{c} DSC\left(\;A,B\;\right)=2*\frac{\left(A\cap B\right)}{\left(A+B\right)}, \end{array}$$

where *A* is the area of the target region of ground truth image acted by the manually segmented images in this paper; *B* is the area of the target region of the result of an automatically segmented image. DSC varies between 0 and 1. The higher it is, the better segmentation accuracy it indicates. According to this fact, the objective function *f* we formulate is

(3) $$\begin{array}{c} DSC=f\left(\;Thresh1,Thresh2\;\right). \end{array}$$

In this way, the optimization problem here can be described as follows: make DSC be as large as possible by selecting appropriate Thresh1 and Thresh2. Since approximate ranges of Thresh1 and Thresh2 can be determined, what we should do next is to find the optimal value from all the possible choices.

The golden section search method is a technique for finding the extremum (minimum or maximum) of a strictly unimodal function by successively narrowing the range of values inside which the extremum is known to exist. The technique derives its name from the fact that the algorithm maintains the function values for triples of points whose distances form a golden ratio known as 0.618. It has been proved that compared to bisection method this value can enable us to obtain an optimal reduction factor for the search interval and minimal number of function calls when searching for the maximum point.

**Figure 8:**
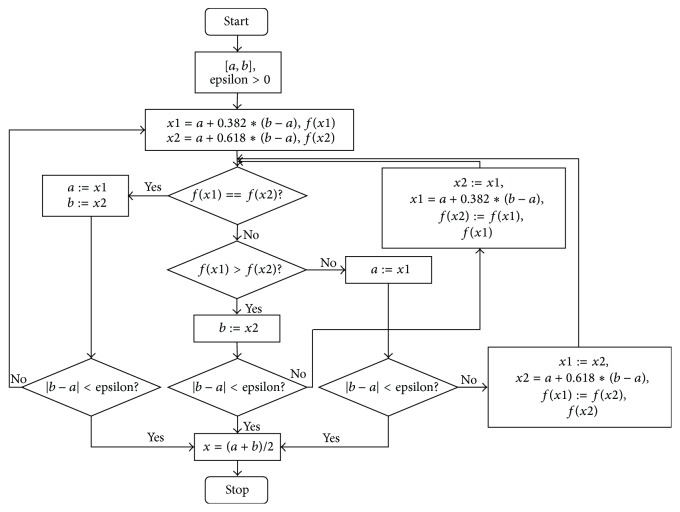
Flowchart of golden section search method.

Assume *f*(*x*) is a unimodal function in search region [*a*, *b*]; the maximum point is *x* and the assumed algorithm precision is epsilon. Let *x*1,  *x*2 be two points in region [*a*, *b*] and *a* < *x*1 < *x*2 < *b*. To use the golden section search algorithm to determine *x*, the following six steps are included (algorithm flowchart is given in Figure 8).

**Step 1 .**

Let [*a*, *b*] be initial search interval and let algorithm precision be epsilon.

**Step 2 .**

Let *x*1 = *a* + 0.382*∗*(*b* − *a*), let *x*2 = *a* + 0.618*∗*(*b* − *a*), and compute *f*(*x*1), *f*(*x*2).

**Step 3 .**

If *f*(*x*1) > *f*(*x*2), *b* : = *x*2, jump to Step 4; if *f*(*x*1) < *f*(*x*2), *a* : = *x*1, jump to Step 5; if *f*(*x*1) = *f*(*x*2), *a* : = *x*1, *b* : = *x*2, jump to Step 6.

**Step 4 .**

If |*b* − *a*| < epsilon, *x* = (*a* + *b*)/2, stop. Otherwise, let *x*2 : = *x*1, *f*(*x*2): = *f*(*x*1), and *x*1 = *a* + 0.382*∗*(*b* − *a*). Compute *f*(*x*1); jump to Step 3.

**Step 5 .**

If |*b* − *a*| < epsilon, *x* = (*a* + *b*)/2, stop. Otherwise, let *x*1 : = *x*2, *f*(*x*1): = *f*(*x*2), and *x*2 = *a* + 0.618*∗*(*b* − *a*). Compute *f*(*x*2); jump to Step 3.

**Step 6 .**

If |*b* − *a*| < epsilon, *x* = (*a* + *b*)/2, stop. Otherwise, jump to Step 2.

In the next section, we will use this method to find the optimal solutions of Thresh1 and Thresh2.

### 2.4. Postprocessing

After threshold segmentation, white blood cells can be roughly segmented (S in Figure 3). At the time, it is still a gray image with some noise, so in this step, binarization is used to convert this resultant image to binary image at first. Then, we use morphological erosion, median filtering (size 15 × 15), and maximum connected region (MCR) extraction operations to fill small holes, connect narrow gaps, and remove small dots as well as the incomplete WBCs in the image. At last, convert the binary image into RGB color image, but this is not a must.

## 3. Results and Discussion

### 3.1. Dataset

**Figure 9:**
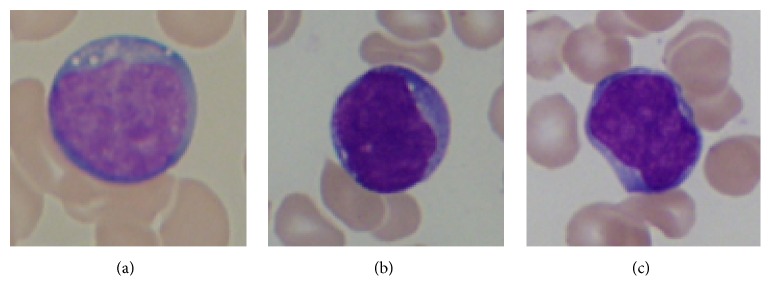
Sample images differ in background color caused by different magnifications being used in image taken.

The proposed method was tested on 130 ALL images taken from ALL_IDB [27], a public and free available dataset provided by Department of Information Technology, specifically designed for the evaluation and comparison of algorithms for segmentation and image classification. The images of the dataset have all been captured with an optical laboratory microscope coupled with a Canon Power Shot G5 camera. For each image in the dataset, the classification of ALL lymphoblast is provided by expert oncologists. ALL_IDB includes two subsets: ALL_IDB1 and ALL_IDB2. The former is composed of 108 images containing about 39000 blood elements taken with different magnifications of the microscope ranging from 300 to 500. So the images in the dataset may differ in background color (Figure 9). The latter is a collection of cropped area of interest of normal or blast cells from ALL_IDB1.

**Figure 10:**
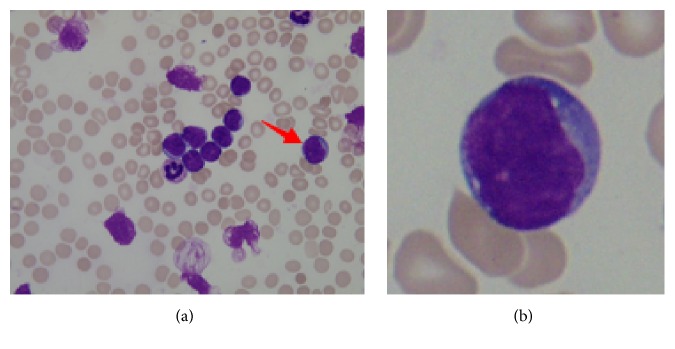
Acute Lymphoblastic Leukemia images from the dataset. (a) ALL_IDB1. (b) ALL_IDB2. Arrow indicates that (b) is cropped from (a).

ALL_IDB2 which contains single WBC in each image is used for testing the performance of our proposal. It has 260 images. The first half is from ALL patients (lymphoblasts) and the last half is from non-ALL patients (normal WBCs). The final task of us is to classify the lymphoblasts into three classes L1, L2, and L3 (Figure 1) for targeted treatment and follow-up, so only the first 130 lymphoblast images of ALL_IDB2 are concerned in this study. But experimental results show that the proposed method also works well for the other 130 images. Two samples from ALL_IDB1 and ALL_IDB2 are shown in Figure 10. Subimage which contains only one WBC can be extracted from multi-WBCs image through a proper method [4, 6, 20].

### 3.2. Experimental Results

We evaluate the performance of our proposed algorithm both visually and quantitatively in this part. To this end, we find the optimal value of Thresh1 and Thresh2 first through the iterative golden section search method described in Section 2. Two doctors are invited to manually segment all test images for the purpose of generating ground truth for evaluation.

#### 3.2.1. Threshold Selection

In the context of this paper, our goal is to find two optimum thresholds to make value of the objective function *f*(Thresh1, Thresh2) (3) be as big as possible. Since there are two variables in this function, the golden section search method needs to be used several times to determine their respective optimum value. Each time, one of them is fixed to find the other variable's optimum value until both of them are unchanged. The idiographic step of this method is given in the following.

**Step 1 .**

Assume that the optimum values of Thresh1 and Thresh2 are, respectively, *t*1, *t*2. To make the procedure operate, initial value of *t*2 is set at 230.

**Step 2 .**

Let Thresh2 = *t*2, *a* = 0.8, *b* = 1.0, epsilon = 0.01, *x* = Thresh1, and *f*(*x*) = mean(DSC(130)) and compute the optimum value of Thresh1 *t*1 through the golden section search method.

**Step 3 .**

Let Thresh1 = *t*1, *a* = 150, *b* = 255, epsilon = 5, *x* = Thresh2, and *f*(*x*) = mean(DSC(130)) and compute the optimum value of Thresh2 *t*2 through the golden section search method. If *t*2 ≠ 230, repeat Step 2 and Step 3. Otherwise, stop.

*Note*

(1) The numbers above, 230, 0.8, 1.0, 0.01, 150, 255, and 5, are selected through a priori knowledge.
(2) mean(DSC(130)) is mean of all 130 test images' DSC value obtained after segmentation on condition Thresh1 = *x*, Thresh2 = *t*2 (in Step 2) or Thresh1 = *t*1, Thresh2 = *x* (in Step 3).

Through the above steps, optimal values of Thresh1 and Thresh2 *t*1,  *t*2 can be determined.

#### 3.2.2. Qualitative Evaluation

**Figure 11:**
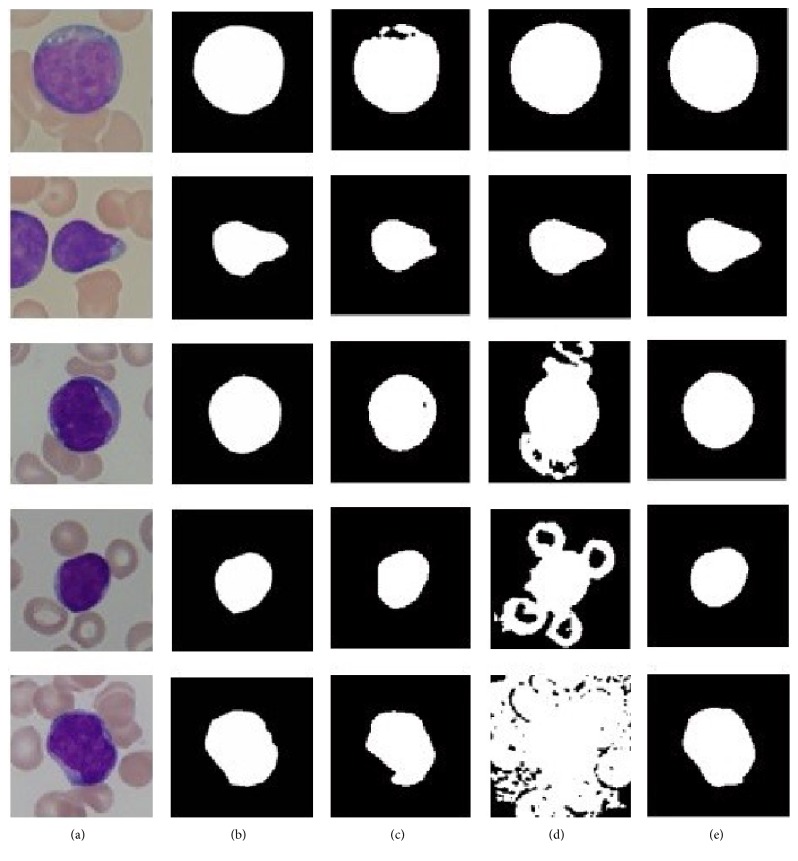
Comparison of the proposed method and the manual method as well as single-threshold methods. (a) Original image. (b) Manual segmentation results (ground truth). (c) M1: RGB color space based single-threshold method. (d) M2: HSV color space based single-threshold method. (e) M3: our proposed method.

Through the above step, the optimal values Thresh1 = 0.9, Thresh2 = 220 are obtained. Segmentation results of the 130 test images are compared with those of manual segmentation as well as two single-threshold methods based, respectively, on RGB [18] and HSV [15] color space. The segmentation result is considered accurate when the autodetected boundary closely matches the manually traced boundary. In Figure 11, we give five test images' segmentation results. Three methods were carried out on the same morphological structure element and median filter sizes. Results present that Method 1 shows a good performance in nucleus segmentation, but the cytoplasm cannot be segmented in some cases (the 1st, 2nd, 4th, and 5th cells). As to Method 2, it segments the WBC perfectly in some cases (the 1st and 2nd cells) but does not in others (the 3rd, 4th, and 5th). On the contrary, our proposed Method 3 performs well in all the cases.

**Figure 12:**
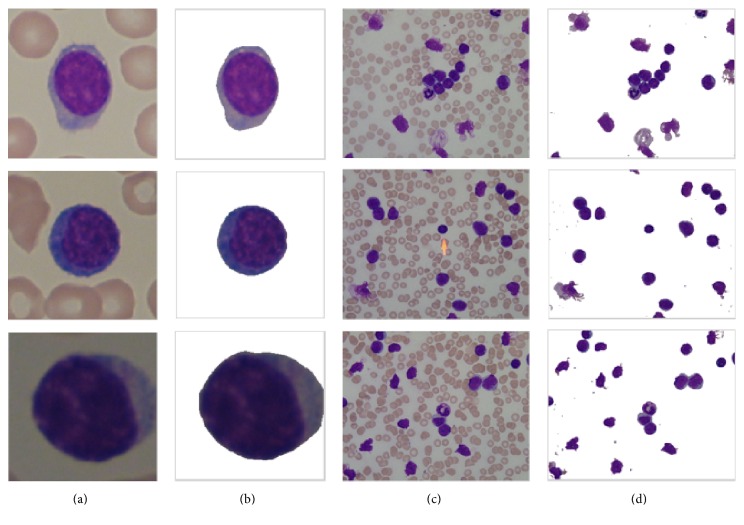
Performance of the proposed method on normal WBCs' and multi-WBCs' segmentation. (a, c) Original images. (b, d) Segmentation results.

It has been shown that the proposed method achieves a high accuracy in segmenting single lymphoblast from microscopic images in Figure 11.  Figure 12 shows that the method also performs well in segmenting normal WBCs in the latter half part of ALL_IDB2 and when multiple WBCs exist in one image (in this case, MCR in postprocessing step should be removed).

#### 3.2.3. Quantitative Evaluation

**Figure 13:**
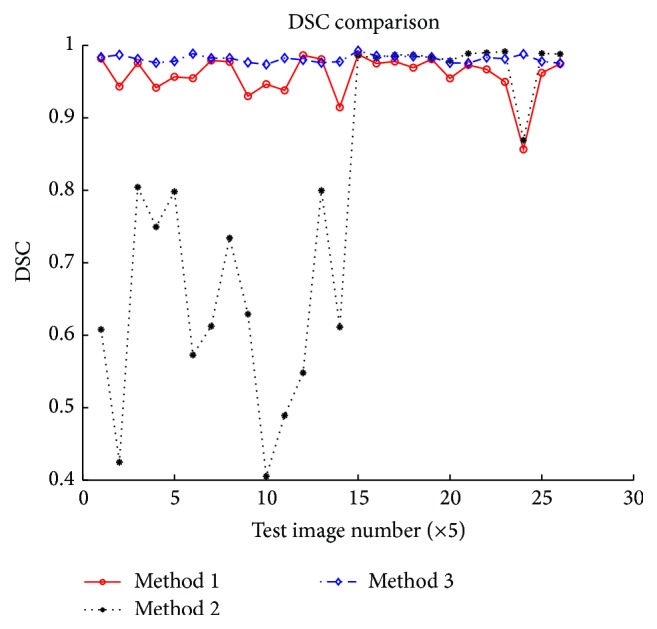
Each test image's DSC value obtained using different segmentation methods. Method 1: RGB color space based single-threshold method. Method 2: HSV color space based single-threshold method. Method 3: our proposed method.

One kind quantitative depiction of segmentation performance is given in Figure 13 where DSC values of the 130 test images are calculated after segmentation with the proposed method and two single-threshold-based methods. It can be seen that DSC values got through our method are higher and stable than the other two methods in most cases. To those images in latter part of all the test images, Method 2 has a good performance but that cannot be extended to whole dataset. As has been said in* dataset* part, images in ALL_IDB are taken with different magnifications, so some of them differ in background color (Figure 9). Thus, H channel image based single-threshold method does not perform well all the time. Mean and standard deviation of the 130 DSCs are given in Table 1. By calculation, we can learn that our method has 2.66% and 26.6% higher accuracy over Method 1 and Method 2, respectively, in terms of DSC mean value metric. Meanwhile, its standard deviation is 64.38% and 93.36% lower than them suggesting our method is also more robust than the other two.

## 4. Conclusion

In this paper, we have proposed a dual-threshold method for segmenting white blood cells from Acute Lymphoblastic Leukemia images. The method effectively combines RGB and HSV color space based single-threshold methods to exploit their complementary strengths. It consists of three main parts: preprocessing, threshold segmentation, and postprocessing. Background and red blood cells of the image are extracted via two different thresholds in segmentation process. The experimental results suggested that an overall segmentation accuracy of DSC ≈ 0.98 can be achieved. As the first step of automatic white blood cell differential system, it shows a good prospect in further WBC feature extraction, ALL classification, and diagnosis.

## Supplementary Material

ALL_IDB2: a public and free available Acute Lymphoblastic Leukemia image database for image processing.

## Figures and Tables

**Table 1:** Mean and standard deviation of all test images' DSC value.

	Method 1	Method 2	Method 3
Mean	0.9542	0.7737	0.9795
Standard deviation	0.0403	0.2163	0.0144

## References

[1] Piuri V., Scotti F. Morphological classification of blood leucocytes by microscope images.

[2] Scotti F. Automatic morphological analysis for acute leukemia identification in peripheral blood microscope images.

[3] Fatichah C., Tangel M. L., Widyanto M. R., Dong F., Hirota K. (2012). Interest-based ordering for fuzzy morphology on white blood cell image segmentation. *Journal of Advanced Computational Intelligence and Intelligent Informatics*.

[4] Cseke I. A fast segmentation scheme for white blood cell images.

[5] Otsu N. (1975). A threshold selection method from gray-level histograms. *Automatica*.

[6] Wu J. H., Zeng P. P., Zhou Y., Olivier C. A novel color image segmentation method and its application to white blood cell image analysis.

[7] Duan J., Yu L. A WBC segmentation methord based on HSI color space.

[8] Jiang K., Liao Q.-M., Xiong Y. (2006). A novel white blood cell segmentation scheme based on feature space clustering. *Soft Computing*.

[9] Dorini L. B., Minetto R., Leite N. J. (2013). Semiautomatic white blood cell segmentation based on multiscale analysis. *IEEE Journal of Biomedical and Health Informatics*.

[10] Arslan S., Ozyurek E., Gunduz-Demir C. (2014). A color and shape based algorithm for segmentation of white blood cells in peripheral blood and bone marrow images. *Cytometry A*.

[11] Dorini L. B., Minetto R., Leite N. J. White blood cell segmentation using morphological operators and scale-space analysis.

[12] Saraswat M., Arya K. V. (2014). Automated microscopic image analysis for leukocytes identification: a survey. *Micron*.

[13] Guo N. N., Zeng L. B., Wu Q. S. (2007). A method based on multispectral imaging technique for white blood cell segmentation. *Computers in Biology and Medicine*.

[14] Zhang C., Xiao X., Li X. (2014). White blood cell segmentation by color-space-based k-means clustering. *Sensors*.

[15] Mohapatra S., Patra D. Automated leukemia detection using hausdorff dimension in blood microscopic images.

[16] Salem N. M. Segmentation of white blood cells from microscopic images using K-means clustering.

[17] Mondal P. K., Prodhan U. K., Al Mamun M. S. (2014). Segmentation of white blood cells using fuzzy C means segmentation algorithm. *IOSR Jornal of Computer Engineering*.

[18] Ko B. C., Gim J.-W., Nam J.-Y. (2011). Automatic white blood cell segmentation using stepwise merging rules and gradient vector flow snake. *Micron*.

[19] Hamghalam M., Motameni M., Kelishomi A. E. Leukocyte segmentation in giemsa-stained image of peripheral blood smears based on active contour.

[20] Di Rubeto C., Dempster A., Khan S., Jarra B. Segmentation of blood images using morphological operators.

[21] Scotti F. Robust segmentation and measurements techniques of white cells in blood microscope images.

[22] Eldahshan K. A., Youssef M. I., Masameer E. H., Mustafa M. A. (2014). Segmentation framework on digital microscope images for acute lymphoblastic leukemia diagnosis based on HSV Color Space. *International Journal of Computer Applications*.

[23] ElDahshan K. A., Youssef M. I., Masameer E. H., Hassan M. A. (2015). Comparison of segmentation framework on digital microscope images for acute lymphoblastic leukemia diagnosis using RGB and HSV color spaces. *Journal of Biomedical Engineering and Medical Imaging*.

[24] Singhal V., Singh P. Correlation based feature selection for diagnosis of acute lymphoblastic leukemia.

[25] Sahidan S. I., Mashor M. Y., Wahab A. S. W. (2008). Local and global contrast stretching for color contrast enhancement on ziehl-neelsen tissue section slide images. *4th Kuala Lumpur International Conference on Biomedical Engineering 2008: BIOMED 2008 25–28 June 2008 Kuala Lumpur, Malaysia*.

[26] Gonzalez R. C., Woods R. E. (2002). Color image processing. *Digital Image Processing*.

[27] Labati R. D., Piuri V., Scotti F. All-IDB: the acute lymphoblastic leukemia image database for image processing.

